# Efficacy and safety of percutaneous nephrolithotripsy in elderly patients: a retrospective study

**DOI:** 10.1186/s12893-022-01830-6

**Published:** 2022-11-16

**Authors:** Jun Liu, Jie Gu, Wenkuan Xu, Cong Tian, Dong Pang, Nanfei Zhang, Yushan Liu, Bo Yang, Xiaobo Huang

**Affiliations:** 1 Department of Urology, Peking University People’s Hospital, 133 Fuchengmen Inner Street, Xicheng District, Beijing, 100034 People’s Republic of China; 2 Peking University Applied Lithotripsy Institute, Peking University, Beijing, 100034 People’s Republic of China; 3 Department of Anesthesiology, Peking University People’s Hospital, Beijing, 100034 People’s Republic of China; 4 Department of Urology, Xishuangbanna Dai Autonomous Prefecture People’s Hospital, Yunnan, 666100 People’s Republic of China; 5 Department of Urology, Mentougou District Hospital, Beijing, 102300 People’s Republic of China; 6 Department of Urology, Shijingshan Teaching Hospital of Capital Medical University, Beijing Shijingshan Hospital, Beijing, 100043 People’s Republic of China; 7 Medical Administration, Beijing Dongcheng First People’s Hospital, Beijing, 100010 People’s Republic of China

**Keywords:** Percutaneous nephrolithotripsy (PCNL), Elderly, Stone free rates, Complications

## Abstract

**Background:**

Percutaneous nephrolithotripsy (PCNL) is difficult to perform for elderly patients; thus, this study aimed to assess its efficacy and safety in elderly patients aged > 70 years, note any associations between outcomes and patient characteristics, and summarize relevant themes and observations.

**Methods:**

Data from patients older than 70 years who had undergone PCNL for upper urinary tract calculi between January 2016 and January 2021 was retrospectively analyzed. Risk factors for postoperative complications and residual stones were analyzed using multivariate logistic regression.

**Results:**

A total of 116 elderly patients underwent 122 PCNL operations, of which six underwent secondary PCNL operations, and all of which were successfully completed. The average age was 74.6 ± 4.3 years; the average stone size and operation time were 3.5 ± 1.8 (1.2–11 cm), and 71.8 ± 34.1 min, respectively. Of the participants, 16 or 13.8% had postoperative complications and 29 (25%) had residual stones after operation. The stone free rate was 75%. Multivariate analysis revealed that an American Score of Anesthesiology III was an independent risk factor for postoperative complications (odds ratio [OR] = 4.453, p = 0.031), and staghorn calculi were independent risk factors for postoperative residual calculi (OR = 31.393, p = 0.001).

**Conclusion:**

PCNL was shown to be safe and effective for elderly patients aged > 70 years. Further, ASA III was an independent risk factor for postoperative complications, and staghorn calculi were independent risk factors for postoperative residual calculi in elderly patients.

## Background

Urolithiasis is a widespread disease that affects all age groups worldwide. An increase in average life expectancy and the disproportionately advancing age of the global population will likely lead to more elderly patients with kidney stones being hospitalized for treatment in the future. According to the European Association of Urology guidelines, percutaneous nephrolithotripsy (PCNL) is the standard treatment method for the treatment of large kidney stones (> 2 cm) and upper ureteral stones [1]. Due to the age-related decline of all organ system functions and the overall decline of physiological reserves, the elderly often ignore routine physical examinations. This also occurs in areas with underdeveloped medical resources. Therefore, when kidney stones need to be treated, they have usually been allowed the time to grow large; Ashley compared PCNL complications in different age groups and found that the incidence of readmission and complications was higher in the elderly than in the young [2]; thus, PCNL for elderly patients is typically challenging [3]. Therefore, in this study, we retrospectively analyzed the efficacy and safety of PCNL in elderly patients (aged ≥ 70 years) to aid urologists to the correctly estimate the outcomes of PCNL in elderly patients.

## Data and methods

### Study population

The data of patients aged ≥ 70 years with upper urinary tract stones who had undergone PCNL between January 2016 and January 2021 in the Urology and Lithotripsy Center of Peking University People's Hospital was retrospectively analyzed with the aim of assessing efficacy and safety. The exclusion criteria were as follows: (1) patients who had undergone bilateral endoscopic lithotripsy in the first-stage operation; (2) patients with serious life-threatening or even more serious systemic diseases, or American Society of Anesthesiologists (ASA) IV-VI status; (3) patients who had undergone urinary diversion surgery; and (4) patients with missing data. A total of 116 patients with detailed clinical data were included, and their general characteristics are shown in Table 1. All patients were informed that their clinical and laboratory data would be used for scientific research, and written consent was obtained prior to the implementation of PCNL. This study was conducted in accordance with the 1964 Declaration of Helsinki and its subsequent amendments, and in accordance with the ethical standards of our hospital’s medical ethics committee.

**Table 1:** General and stone related information of elderly patients

Parameters	No.(%)	Mean ± SD
Age	116 ()	74.6 ± 4.3
70–75	68 (58.6)	
75–80	35 (30.2)	
> 80	13 (11.2)	
Sex (male/female)	69/47	
BMI		24.8 ± 3.4
ASA		
I/II	95	
III	21	
Medical comorbidities		
Hypertension	66 (56.9)	
Diabetes	49 (42.2)	
Coronary heart disease	21 (18.1)	
Pre-operative creatinine (μmol/L), mean ± SD		90.0 ± 44.8
Kidney left/rignt	72/44	
Stone location		
Non-staghorn	51 (44.0)	
Staghorn	65 (56.0)	
Stone size(mm)		3.5 ± 1.8
≤ 2 cm	24 (20.7)	
2–4 cm	61 (52.6)	
> 4 cm	31 (26.7)	
Hydronephrosis		
Non or slight	25 (21.6)	
Moderate or severe	91 (78.4)	
Staging operation	6 (5.2)	

Data related to the following variables were collected and analyzed: demographics, comorbidity, body mass index (BMI), ASA grade, stone size, operation time (in minutes), blood transfusion, stone free rate (SFR), surgical complications (intraoperative, perioperative, and postoperative periods recorded according to the improved Clavien classification system), and length of hospital stay[4].

Staghorn calculi are defined as upper urinary tract calculi involving the renal pelvis and extending to at least two renal calices.

The stone-free state was defined as the disappearance of kidney stones or the presence of fragments smaller than 4 mm according to postoperative imaging examination.

### Percutaneous nephrolithotomy

All patients received combined spinal epidural anesthesia. In the lithotomy position, a 5-French (Fr) ureteral catheter (C.R. Bard, New Providence, New Jersey, USA) was placed on the affected side. The patients were placed in the prone position, and percutaneous access was obtained under ultrasound guidance. Then, using the guidewire, the pathway was expanded through the matched peel sheath (Cook Medical, Bloomington, IN, USA). Renal pathways of 16–24F were established according to each situation. A 14 and 20.8 Fr rigid nephroscope (Richard Wolf, Knittlingen, Germany) and ultrasonic lithotripsy were used for stone fragmentation and removal [4], and an ultrasonic removal system used for clearing the crushed stone (Huifu kang Co. Beijing, China). At the end of the operation, a 6F double J stent and nephrostomy tube were placed for drainage, retained in the body for 4 weeks and 3 days, respectively. Kidney, ureter, and bladder and renal ultrasounds were performed in all patients on the first postoperative day to evaluate residual stones.

Following PCNL, the improved Clavien classification system was used to classify complications [5].

### Statistical methods

SPSS 20.0 (IBM Corp., Armonk, NY, USA) was used for statistical analysis. Data are presented as the mean ± standard deviation (SD) or median and range. Multivariate logistic regression analysis was used to analyze risk factors, and statistical significance was set at p < 0.05.

## Results

**Fig. 1:**
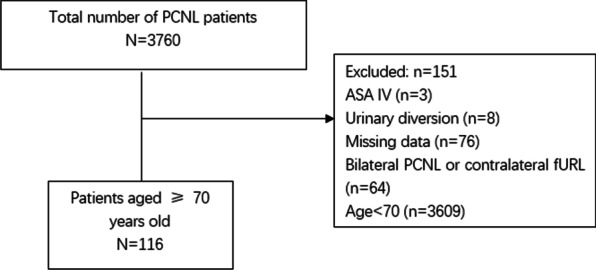
Flow chart

**Fig. 2:**
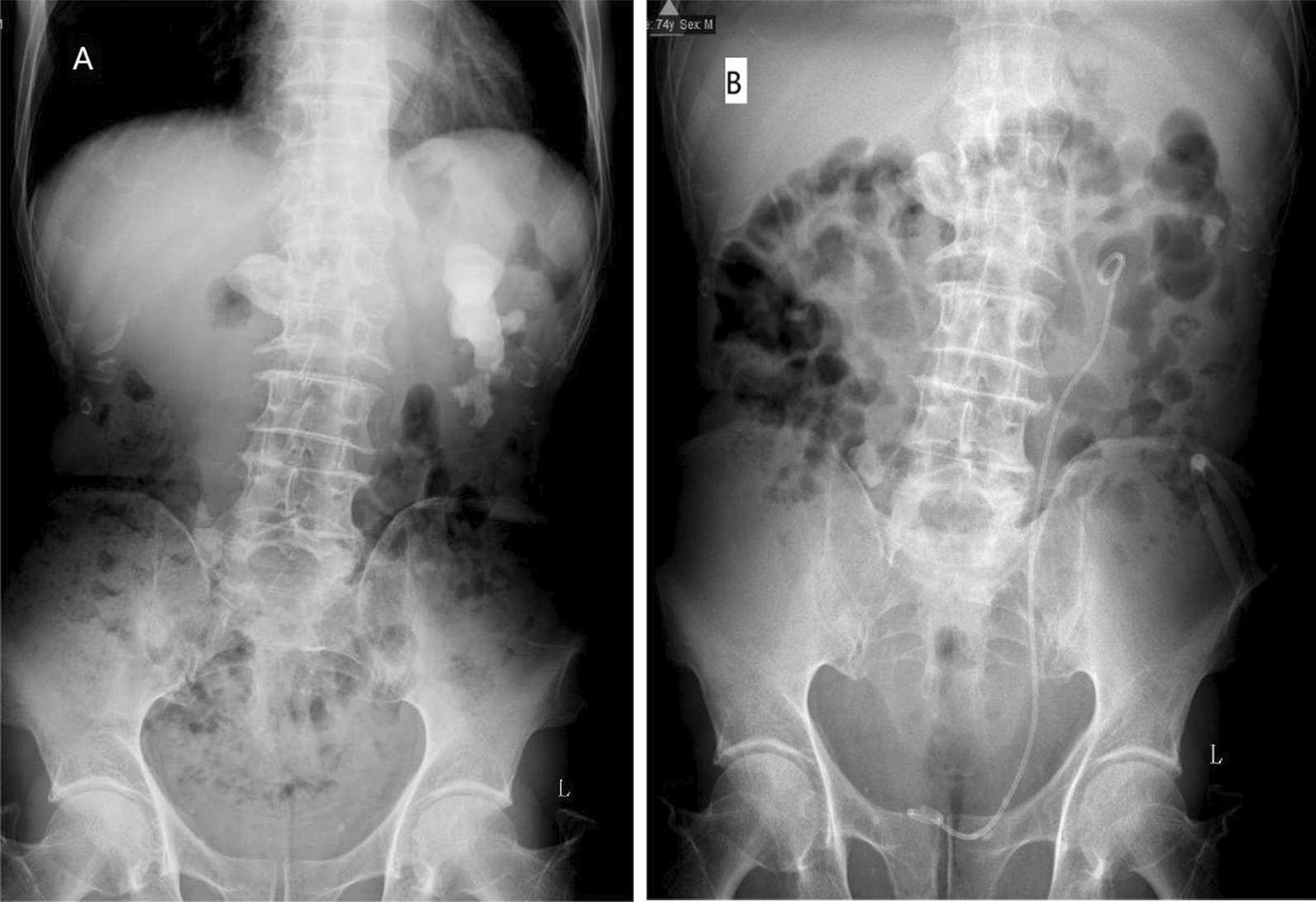
**A** A 74 year old male with staghorn calculi in the left kidney was treated with single channel, 22F, percutaneous nephrolithotripsy. **B** Most of the calculi were cleared by KUB after operation, and a small amount of residual calculi were found in the kidney. KUB = Kidney, Ureter, and Bladder X-ray

Detailed clinical data of 116 consecutive patients were collected (Fig. 1). Of the 116 patients that completed 122 PCNL operations, six underwent second-stage PCNL operations. Among them, 69 were male and 47 were female. The average age of the patients was 70–88, the average age was 74.6, and the average stone size was 3.5 cm. Table 1 shows the general and stone-related information of the patients. Figure 2 shows the stone characteristics of one patient, a 74-year-old man.

The operation data and prognosis of the patients are summarized in Table 2. The average operation time was 71.8 min, postoperative hospital stay 5.2 days, and postoperative SFR 75%. There were no deaths during the perioperative period, and the probability of postoperative complications was 13.8%. Among the patients, three had Clavien IV complications, who were transferred to the ICU for treatment due to pneumonia, heart failure, and septic shock, respectively; however, the prognosis was good.

**Table 2:** PCNL operation and postoperative complications

Parameters	No. (%)	Mean ± SD
Operation time		71.8 ± 34.1
< 60 min	40 (34.5)	
≥ 60 min	76 (65.5)	
No. channel		
Single	99 (85.3)	
Multiple	17 (14.7)	
Channel size		
16F	14 (12.1)	
18–22F	36 (31.0)	
24F	66 (56.9)	
Postoperative hemoglobin drop (g/L)		12.9 ± 6.8
Postoperative hospital time(d)		5.2 ± 3.6
Initial SFR	87/116 (75.0)	
Stone composition	47 (40.5)	
Calcium oxalate	39	
Magnesium phosphate	5	
Uric acid	9	
Singular stone composition	29	
Mixed stone composition	18	
Surgical complications, n (%)	16 (13.8)	
Fever (Clavien I/II)	11	
Obstruction (Clavien III)	2	
ICU (Clavien IV)	3	
Pneumonia	1	
Heart failure	1	
Septic shock	1	

In our multifactor analysis of factors related to the postoperative complications of PCNL, ASA grade III was shown as an independent risk factor for postoperative complications in elderly patients (OR = 4.453, p = 0.031) (Table 3). We also conducted a multivariate analysis of residual stones that revealed staghorn stones as an independent risk factor for residual stones after PCNL (or = 31.393, p = 0.001) (Table 4).

**Table 3:** Risk factors of postoperative complications in patients from multivariate analysis

	OR	95% CI	P value
Female/male	0.752	0.207–2.735	0.666
BMI (> 28)	0.479	0.093–2.461	0.378
ASA grade III	4.453	1.147–17.290	0.031
Intraoperative hypotension	2.253	0.682–7.441	0.183
Preoperative renal insufficiency	0.488	0.093–2.573	0.398
Preoperative urinary tract infection	0.534	0.111–2.564	0.433
Staghorn calculus	3.080	0.754–12.574	0.117
Hydronephrosis	0.331	0.092–1.187	0.090
OR time (> 60 min)	0.622	0.181–2.144	0.452
Multichannel surgery	0.597	0.107–3.336	0.556

**Table 4:** Risk factors of postoperative residual stone from multivariate analysis

	OR	95% CI	P value
BMI (> 28)	1.008	0.300–3.384	0.990
Operation time	1.356	0.388–4.745	0.634
ASA	0.977	0.262–3.640	0.972
Staghorn calculus	31.394	3.943–249.942	0.001
Hydronephrosis	2.540	0.686–9.405	0.163

## Discussion

As a retrospective study with a large sample size in a single center, this was the largest PCNL study of its kind to date. In it, we primarily assessed the characteristics, safety, and effectiveness of PCNL in elderly patients over 70 years of age. Based on the results, we draw the following conclusions. First, PCNL is safe and effective in the treatment of kidney stones in elderly patients over 70 years. Second, ASA III was an independent risk factor affecting surgical complications, and staghorn calculi was an independent risk factor affecting postoperative SFR.

In China, the development of medical technology varies among different cities. Many elderly people have poor medical awareness and often do not undergo annual physical examinations. Therefore, treatment of kidney stones in the elderly has become a difficult problem for urologists. According to statistics, the incidence of kidney stones worldwide is approximately 1%; however, in most developed countries, it is approximately 10%. The increase in the aging population will likely lead to more elderly patients with hospitalized for kidney stone treatment. However, owing to the risk of urinary tract infection and renal function damage, conservative treatment of kidney stones in the elderly may not be applicable [11].

With the development of technology, namely improved optical and endoscopic quality, there are many options for the treatment of kidney stones in the elderly. However, PCNL remains an effective treatment for larger stones [12]. Although there is evidence to prove the effectiveness of PCNL surgery, a 20–83% rate of complications has been reported in the literature, including bleeding requiring blood transfusion, pleural injury, and colon injury [13]. Although staged flexible ureteroscopic lithotripsy (FURL) can also handle large kidney stones, Knoll et al. confirmed that patients with kidney stones with an average stone size of 19 mm receiving FURL require two operations [14]. In our study, the average stone size was 3.5 cm, and the proportion of stones > 4 cm reached 26.7%. Of these, the largest kidney stone diameter was 11 cm (Fig. 2). However, staged FURL may not be advisable in the elderly population as repeated exposure to anesthesia is problematic for elderly patients.

The elderly have poor cardiopulmonary function reserve, and the incidence rates of diabetes, hypertension, coronary artery disease, cerebrovascular disease, peripheral vascular disease, tumor, chronic kidney disease, and the related complications are high [13]. Further, many elderly patients use anticoagulants for the treatment of cardiovascular and other chronic diseases. These factors lead to the deterioration of cardiopulmonary reserve, increased risk of surgical anesthesia, and increased risk of bleeding and septic complications, which may be fatal in the elderly population. The incidence of complications in this study was 13.8%, which was similar to that reported in the literature [3, 6–10] (Table 5). Through a multifactor analysis of factors related to complications, we found that ASA grade III was an independent risk factor for complications following PCNL in the elderly. Similarly, Hackett previously confirmed that ASA grade is an independent risk factor for related medical complications after surgery [15]. Therefore, for elderly patients with ASA grade III, we must carefully evaluate the risks and benefits of surgery in conjunction with anesthesiologists.

**Table 5:** Comparison between conditions of elderly patients undergoing PCNL operations as reported in the literature

Study	Country	Number of cases	Inclusion of age criteria	average age	Stone size	Operation time	SFR	Complications
Sahin (2001) [2]	Turkey	27	60	65.8	(1077.92 mm^2^	101.4 min	89%	18.5%, Perirenal hematoma 1 Fever without bacteremia 4
Abedali (2019) [6]	USA	59	80	83	2.2 ± 1.9	–	72.7%	22.0%, Transfusion 10.2%, 6 respiratory distress 8.5%,5 Sepsis3.4%,2
Iqbal (2021) [7]	Pakistan	79	60	63.4	449 ± 163mm2	151.3	(79.74%	21.5%, Transfusion 1 Sepsis 2
Gupta (2020) [8]	India	50	65	66.8	2.2 ± 0.6	58.54 ± 18	94	20.0%, transfusion 4 Fever 6
Okeke (2012) [9]	The Netherlands	334	70	74.7	465.0	85.7	83.3	Minor complications (%) (Clavien I and II) 14.8 Major complications (%) (Clavien III–V) 5.1
Nakamon (2013) [10]	Thailand	61	65	70.7	4.1 cm	52.6	70.49%	(13.11%), Blood transfusion 4 (6.55%) Sepsis 4 (6.56%)
Our study	China	116	70	74.6	3.5 ± 1.8	71.8 ± 34.1	75	13.8% Fever (Clavien I/II) 11 obstruction (Clavien III) 2 ICU(Clavien IV) 3 Pneumonia 1 Heart failure 1 Septic shock 1

Staghorn calculi are always difficult to detect in PCNL, as their shape is complex and involves multiple renal calices, which may increase the time requirement for lithotripsy and stone removal during surgery as well as the possibility of multiple surgical channels [16]. In our study, antler calculi were found to be independent risk factors for postoperative residual calculi. Owing to their complexity, we believe that the operation time should not be prolonged or the number of operation channels excessively increased in order to pursue complete stone removal. Patient safety is the basis for completion of the operation.

Through this summary of surgical experience over the last 5 years, we have summarized the following points. (1) For patients with more basic diseases, intraspinal anesthesia can help the operation with effective completion, can help avoid the risk of pulmonary infection or atelectasis caused by general anesthesia, and is suitable for postoperative management of the elderly [17]. (2) Of our patients, 56.9% required a 24F percutaneous renal channel. This is convenient for stone removal and can help the avoidance of infection complications caused by an increase in renal pelvic pressure during continuous perfusion. (3) We recommend the use of ultrasonic lithotripsy; therefore, during the lithotripsy process, the pressure in the kidney can be maintained at a relatively low level and stones can be removed quickly. If hard stones are encountered, they can also be crushed in combination with pneumatic lithotripsy or a holmium laser. (4) For patients with large stones, staged surgery may be used to avoid surgical complications due to the long operation time. In this study, six patients underwent staged surgery, and all completed the operation well.

This study had some limitations. First, owing to its retrospective design, there may have been a selection bias. Also, since the procedure was uniform, the outcomes from different modifications of PCNL were not analyzed. However, this study provides valuable evidence for the use of PCNL in patients aged > 70 years. To cope with the increasing number of elderly people, it is necessary to conduct a prospective study on elderly patients with a larger sample size.

In conclusion, it is safe and effective to provide PCNL for elderly individuals aged > 70 years. In the elderly, ASA III is an independent risk factor for postoperative complications and staghorn calculi are independent risk factors for postoperative residual calculi. These findings should help urologists to perform PCNL more safely in elderly patients.

## Data Availability

The datasets used and analyzed during the current study are available from the corresponding author upon reasonable request.

## Abbreviations

PCNL: Percutaneous nephrolithotomy
BMI: Body mass index
OR: Odds ratio
ASA: American Score of Anesthesiology
SFR: Stone free rates
KUB: Kidney, Ureter, and Bladder X-ray
